# Polyomavirus BK Genome Comparison Shows High Genetic Diversity in Kidney Transplant Recipients Three Months after Transplantation

**DOI:** 10.3390/v14071533

**Published:** 2022-07-14

**Authors:** Olga Mineeva-Sangwo, Joan Martí-Carreras, Evert Cleenders, Dirk Kuypers, Piet Maes, Graciela Andrei, Maarten Naesens, Robert Snoeck

**Affiliations:** 1 Laboratory of Virology and Chemotherapy, Rega Institute, Department of Microbiology, Immunology and Transplantation, KU Leuven, BE3000 Leuven, Belgium; olga.mineevasangwo@kuleuven.be; 2 Zoonotic Infectious Diseases Unit, Laboratory of Clinical and Epidemiological Virology, Rega Institute, Department of Microbiology, Immunology and Transplantation, KU Leuven, BE3000 Leuven, Belgium; joan.marti@kuleuven.be (J.M.-C.); piet.maes@kuleuven.be (P.M.); 3 Nephrology and Renal Transplantation Research Group, Department of Microbiology, Immunology and Transplantation, KU Leuven, BE3000 Leuven, Belgium; evert.cleenders@kuleuven.be (E.C.); dirk.kuypers@kuleuven.be (D.K.); maarten.naesens@kuleuven.be (M.N.); 4 Department of Nephrology and Renal Transplantation, University Hospitals Leuven, BE3000 Leuven, Belgium

**Keywords:** BK polyomavirus, kidney transplantation, viral load, viremia, PVAN, genetic diversity, next generation sequencing, complete genome assembly, BKAnaLite pipeline

## Abstract

BK polyomavirus (BKPyV) is a human DNA virus generally divided into twelve subgroups based on the genetic diversity of Viral Protein 1 (VP1). BKPyV can cause polyomavirus-associated nephropathy (PVAN) after kidney transplantation. Detection of BKPyV DNA in blood (viremia) is a source of concern and increase in plasma viral load is associated with a higher risk of developing PVAN. In this work, we looked for possible associations of specific BKPyV genetic features with higher plasma viral load in kidney transplant patients. We analyzed BKPyV complete genome in three-month samples from kidney recipients who developed viremia during their follow-up period. BKPyV sequences were obtained by next-generation sequencing and were de novo assembled using the new BKAnaLite pipeline. Based on the data from 72 patients, we identified 24 viral groups with unique amino acid sequences: three in the VP1 subgroup IVc2, six in Ib1, ten in Ib2, one in Ia, and four in II. In none of the groups did the mean plasma viral load reach a statistically significant difference from the overall mean observed at three months after transplantation. Further investigation is needed to better understand the link between the newly described BKPyV genetic variants and pathogenicity in kidney transplant recipients.

## 1. Introduction

The successful introduction of newer and more potent immunosuppressive agents improved the degree of graft survival in renal transplant recipients [1] but led to an increased number of polyomavirus-associated nephropathy cases (PVAN). An important cause of renal failure early after kidney transplantation, PVAN is mainly induced by the human BK polyomavirus (BKPyV) [2].

BKPyV is a small double-stranded DNA virus. The BKPyV genome is approximately 5-kb long and consists of two highly conserved regions encoding for early (LTag, sTag) and late (Agno, VP1, VP2, VP3) proteins, separated by a non-coding control region, NCCR [3]. BKPyV can be divided into four different subtypes (I, II, III and IV) and/or twelve subgroups (Ia, Ib1, Ib2, Ic, II, III, IVa1, IVa2, IVb1, IVb2, IVc1 and IVc2). BKPyV genotyping has historically been based on the VP1 gene, however, numerous subtype/subgroup-specific polymorphic sites have also been identified throughout the entire coding region of the BKPyV genome [4,5,6]. Subtype I can be found in all geographic regions, followed by subtype IV. Subtypes II and III are rare [7]. In vitro studies suggest that BKPyV subtypes may differ in cell tropism and pathogenic potential [8,9], and there might be functional differences among different subtypes/subgroups [10,11,12]. The NCCR of BKPyV contains a promoter/enhancer for the transcription of both the early and late coding regions, as well as an origin of replication [13]. Based on the composition of the NCCR, BKPyV can be classified as an archetype or a rearranged form [14]. The archetypal NCCR has a linear O-P-Q-R-S structure of 142 (O), 68 (P), 39 (Q), 63 (R) and 63 (S) base pairs [13,15]. Duplications and/or deletions of complete or partial sequence blocks are associated with the emergence of rearranged BKPyV variants [13]. It was proposed that NCCR variation may influence the replication rate of BKPyV and therefore may play an important role in disease development in kidney transplant recipients [16].

After the primary infection, BKPyV remains in the kidney as a prominent source of latent infection [17] and can be shed episodically in the urine in both healthy and immunosuppressed individuals [18,19]. In kidney transplant recipients, the detection of BKPyV in blood (viremia) is associated with further spread of infection [20] and increased risk of developing PVAN [21]. Screening for BKPyV replication is recommended before significant functional impairment can occur in the allograft [22]. BKPyV replication can be monitored using the viral load metric obtained by polymerase chain reaction (PCR). Studies on viral load dynamics show that urine BKPyV load of >1 × 10^7^ copies/mL correlate with the onset of viremia while an increase in the blood PCR value to >1 × 10^4^ copies/mL is associated with an increased rate of histologically confirmed PVAN in kidney transplant recipients [23,24,25,26].

In this work, we analyzed the entire genome of BKPyV in samples collected three months after kidney transplantation for the presence of specific genetic features that may be associated with higher plasma viral loads. We were able to identify genetically distinct BKPyV variants based on rearrangements in the non-coding control region (NCCR), polymorphisms in the VP1 gene and amino acid changes in the entire protein-coding region. We further investigated the association of any of the variants with high plasma viral loads observed three months after kidney transplantation by comparing the mean plasma viral load of the groups with that of the overall mean of all the samples.

## 2. Materials and Methods

### 2.1. Study Cohort and Sample Collection

**Figure 1:**
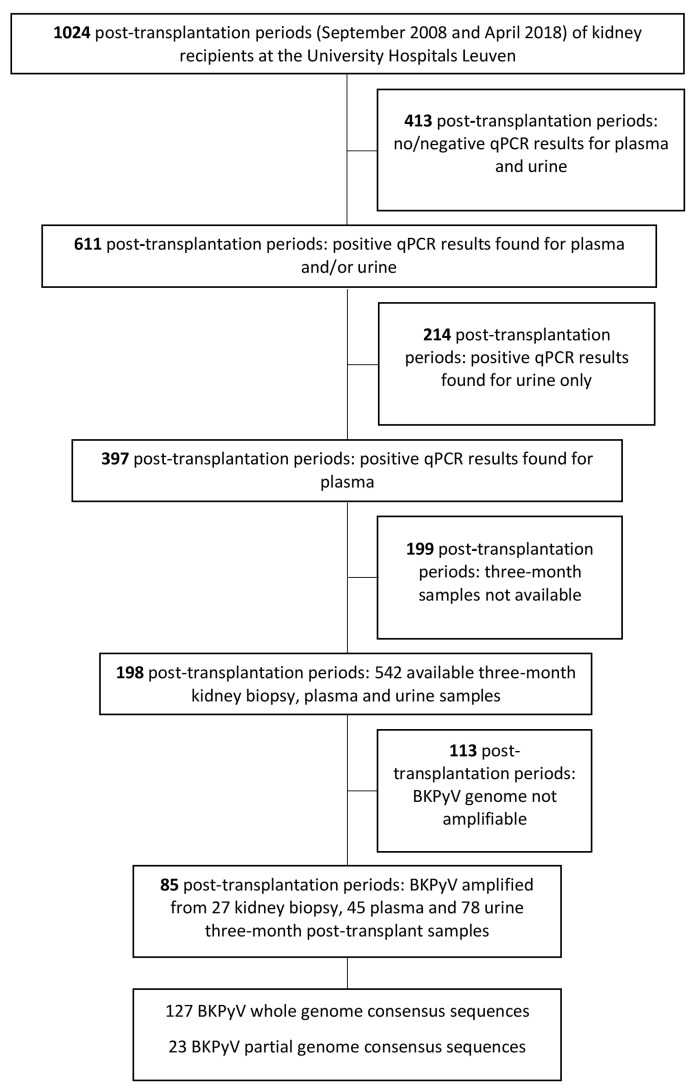
Sample selection and BKPyV genome amplification in kidney transplant patients with positive qPCR results observed in plasma during the post-transplant follow-up period.

The study enrolled patients of the University Hospitals Leuven (Leuven, Belgium) who received a kidney allograft in the period between September 2008 and April 2017. Samples were taken from patients with a plasma viral load of >2.7 log10 copies/mL (BKPyV/JCPyV quantitative PCR; quantification range 2.7–7.7 log10 copies/mL) detected at any time in the post-transplantation period from September 2008 to April 2018. Since some patients underwent more than one transplantation in the indicated period, we also specify (where applicable) the total number of patients. We analyzed 1024 post-transplantation periods (Figure 1). Viral DNA was detected in plasma samples (>2.7 log10 copies/mL) in 397 post-transplantation periods (394 patients). Three-month post-transplantation samples were selected for further analysis and included plasma, urine and kidney biopsy samples taken at the same post-transplant time-point as part of the routine clinical follow-up of kidney transplant recipients. In total, 542 samples were available for the analysis. The average time interval between the transplantations and the samples was 95 days. The study was approved by the Ethics Committee of the University Hospitals Leuven (S53364 and S61201) and all patients consented to using their clinical data for study purposes.

### 2.2. Immunosuppressive Therapy

The standard immunosuppressive regimen consisted of tacrolimus, mycophenolate-mofetil and corticosteroids, with additional basiliximab induction therapy in patients at increased immunological risk. Changes in immunosuppression were conducted at the discretion of the treating physician.

### 2.3. DNA Isolation

Total DNA was isolated from kidney allograft biopsies using the Allprep DNA/RNA/miRNA Universal Kit (Qiagen Benelux BV, Hulsterweg, The Netherlands) on a QIAcube instrument (Qiagen Benelux BV). From urine and plasma samples, DNA was extracted using QIAamp DNA Blood Midi Kit (Qiagen, Benelux BV) according to the manufacturer’s protocol. To obtain more concentrated extracts from larger sample volumes (600 µL), DNA was eluted in 150 µL of buffer.

### 2.4. BKPyV Genome Amplification

BKPyV genome was amplified using Invitrogen Platinum SuperFi PCR Master Mix (Thermo Fisher Scientific, Brussel, Belgium). Two sets of primers were used to amplify the whole genome of BKPyV. The primers were designed by selecting a highly conserved region of human BK virus genome (GenBank accession number V01108.1, Dunlop strain). Primer specificity was tested using Blast [27] with default parameters. The first set of primers was used to amplify a portion of the BKPyV genome (hereafter, amplicon 1) starting at nucleotide 853 (coding region for VP2) and ending at 3255 (coding region for LTag). The second set of primers was used to amplify the rest of the genome (hereafter, amplicon 2) starting at nucleotide 2868 and ending at 1222 (Figure S1). Amplicon 2 also contained BKPyV NCCR. PCR products were confirmed by agarose gel electrophoresis and purified using AMPure XP SPRI beads (Beckman). The primer sequences used are listed in Table S1.

### 2.5. Next-Generation Sequencing (NGS)

PCR amplicons were sequenced using Illumina MiSeq technology. The amplicons were quantified with a Qubit fluorometer (Thermo Fisher Scientific) and library construction with indexes was performed using 1 ng DNA of each sample with Nextera XT kits (Illumina). Libraries were quantified (Qubit) and then pooled in equimolar amounts (2 nM). PhiX Control v3 library was added to the libraries at 5% of the total volume. The library pool was subjected to the sequencing using Miseq v.2 system with the paired-end (2 × 150 bp) workflow.

### 2.6. BKPyV Genome Sequence Reconstruction

BKPyV complete genome sequence was reconstructed using a new bioinformatics pipeline, BKAnaLite. Firstly, both forward and reverse (hereafter, R1 and R2, respectively) sets of reads from amplicon 1 and 2 were merged. Then reads were trimmed based on possible adapter sequencing content, length and quality using BBDuk (minlength = 150, trimq = 30, qtrim = w, minavgquality = 20) [28]. Posteriorly, read coverage was 2-step k-mer normalized using BBNorm (first target = 99,999,999, min = 500, passes = 1, then target = 1000, ecc = t, keepall passes = 1, bits = 16 prefilter), ensuring that genomic regions where amplicons overlap, have comparable coverage to the rest of the genome [28]. Additionally, homogeneous read downsampling through normalization ensured a balanced de novo genome assembly. De novo genome reconstruction was conducted by IVA [29]. Blast was used to extract the complete genome from the assembly by length and, to orient and circularize it to match the reference (V01108.1). BKPyV genomes were polished to generate a consensus from the viral population by mapping back all trimmed reads to the genome using BWA-MEM and Pilon (correcting for small indels introduced during assembly) [30,31]. The pipeline, which also includes a variant-calling algorithm, is available on https://github.com/joanmarticarreras/BKAnaLite (accessed on March 2021). The sequences (*n* = 127) have been deposited in GenBank under accession numbers OM938824–OM938950.

A partial DNA sequence of the BKPyV genome was obtained (CLC Genomics Workbench v12.0.3 software package, Qiagen Benelux, The Netherlands) when only one of the two amplicons (amplicon 1 or 2) was available. The quality control of the reads was performed using the QC for Sequencing Reads tool. Reads were trimmed using the Trim Reads tool with default parameters. Thereafter, the trimmed reads were mapped against the reference to obtain a consensus sequence. The sequence of BKPyV NCCR was determined using the Sanger sequencing method. The NCCR was first amplified from amplicon 2 using two primers (Table S1). The resulting amplicon was then sequenced using an ABI Prism 3730XL DNA Analyzer (Thermo Fisher Scientific). Bi-directional sequencing was performed with the Big Dye Terminator v3.1 sequencing kit (Thermo Fisher Scientific) following the manufacturer’s recommendations. Chromatograms were analyzed with SeqScape v2.7 software (Applied Biosystems, Foster city, CA, USA) to obtain the consensus sequence.

### 2.7. BKPyV and NCCR Typing

The genotyping was performed using the recently developed automated BKTyper v0.2 tool [32] based on the VP1 gene. The same tool was used to define BKPyV NCCR structure.

### 2.8. BKPyV Amino Acid Changes Analysis

The analysis was performed using BKPyV VP1, VP2, VP3, LTag, sTag and Agno amino acid sequences derived from BKPyV whole genome consensus sequences. The amino acid sequences of each BKPyV protein were aligned separately using MAFFT (v7.471) with default parameters [33] in order to compare and determine amino acid replacement sites among them. The numbering of amino acid follows that of V01108.1.

### 2.9. Statistical Analysis

Data were analyzed using R software (R Development Core Team, R version 4.0.4 and R studio version 1.3.1093, https://www.R-project.org/), and corresponding plots were constructed with the R package ggplot2 (Wickham, version 3.3.3). Association between BKPyV and viral load in plasma was assessed using a parametric survival model with the assumption of normally distributed residuals. Plasma viral loads <2.7 log10 copies/mL were treated as left-censored observations [34]. For all performed tests, differences were considered statistically significant at *p* < 0.05.

## 3. Results

### 3.1. Study Cohort and Sample Collection

The present study aimed at analyzing BKPyV genome in three-month post-transplantation samples of kidney transplant recipients. The selected samples were collected from 394 patients (397 post-transplantation periods) who showed viral DNA in the blood (>2.7 log10 copies/mL) at some point in time during the post-transplantation observation (Figure 1). We were able to amplify BKPyV DNA in 150 samples from 85 patients. In 46 patients, BKPyV DNA was amplified in two or three samples. In the other 39 patients, it was amplified in only one sample. We were able to obtain the complete sequence of BKPyV genome in 127 samples and partial sequence in 23 samples (Figure 1).

We also checked the available plasma samples for a possible presence of JCPyV DNA. We targeted two different regions of the JCPyV genome using two set of primers (Table S1) but were unable to amplify JCPyV DNA in any of the plasma samples.

### 3.2. BKPyV VP1 Genotype and Plasma Viral Load 

**Figure 2:**
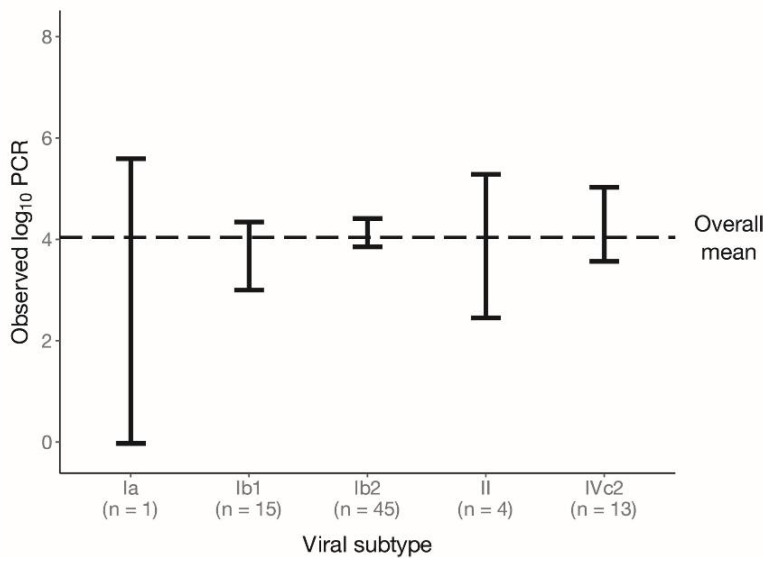
Distribution of the observed plasma BKPyV load values for each subtype/subgroup. The black error bars represent the mean ± (1.96× standard error) for each subtype/subgroup. The black dashed line shows the mean of all observations with an identified subtype/subgroup (*n* = 78). Viral load <2.7 log10 copies/mL was replaced by 1.35 log10 copies/mL.

We genotyped BKPyV based on the VP1 gene from 140 samples: 45 plasma, 72 urine and 23 kidney biopsy samples (78 patients). Our analysis showed the prevalence of subtype I (*n* = 109, 77.8%), and more specifically that of subgroups Ib2 (*n* = 85, 60.7%), Ib1 (*n* = 23, 16.4%) and Ia (*n* = 1, 0.7%). In the remaining samples (*n* = 31, 22.1%), subgroup IVc2 (*n* = 26, 18.6%) and subtype II (*n* = 5, 3.6%) were found. We compared, where possible, BKPyV subtype/subgroup in plasma and other samples from the same patient (*n* = 42) and found that it was the same across the samples. This observation allowed us to analyze the subtype/subgroup association with plasma viral load including samples from 45 patients with a known subtype/subgroup in plasma as well as samples from 33 patients with a subtype/subgroup obtained only from urine and/or kidney. There was one patient who had no plasma sample, subtype II in kidney biopsy and subgroup Ib2 in urine samples. In this case, we included only one of the two samples in the analysis. We compared the mean of the plasma viral load observed in subtype II (*n* = 4) and subgroups Ia (*n* = 1), Ib1 (*n* = 15), Ib2 (*n* = 45) and IVc2 (*n* = 13) with the overall mean of the plasma viral load in the samples from all 78 patients. We did not observe a statistically significant difference in plasma viral load in any of the subtypes/subgroups (Figure 2; Table S2).

### 3.3. BKPyV Amino Acid Diversity and Plasma Viral Load

We further extended our analysis and examined the entire protein-coding region of BKPyV in the samples. We included in the analysis complete amino acid sequences of BKPyV obtained in 127 samples, specifically 43 plasma, 63 urine and 21 kidney biopsy samples (72 patients). We described each sample based on amino acid substitutions identified in BKPyV Agno, sTag, LTag, VP1, VP2 and VP3 proteins (Table 1) and compared amino acid changes in plasma samples with those found in other samples from the same patient (*n* = 40).

**Figure 3:**
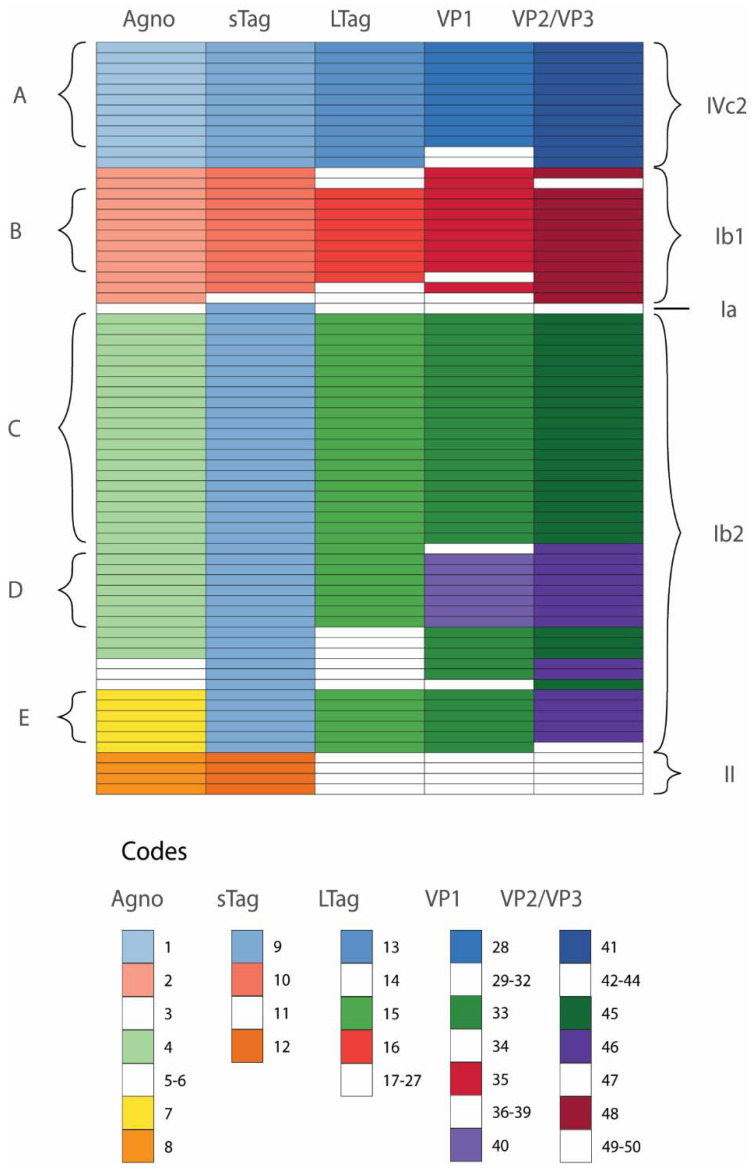
Visual representation of BKPyV amino acid diversity in three-month post-transplant samples from 72 patients. Each row represents one sample. One patient had BKPyV VP1 subtype II in kidney biopsy sample and BKPyV VP1 subgroup Ib2 in urine sample. In this case, only one of the two samples was included. Two other patients showed amino acid differences in VP1 protein in their urine and plasma samples; only one sample per patient was included in this case. Identical amino acid sequences (codes) of Agno, sTag, LTag, VP1 or VP2/VP3 found in two or more samples are brightly colored; those found in fewer than two samples are white.

In most cases (*n* = 38; 95%) we found the same amino acid changes in all six BKPyV proteins. Based on this observation we included in our further analysis samples from 29 patients with no BKPyV amino acid sequences from plasma. We included only one of two samples from two patients who showed differences in amino acid sequences from plasma and urine. The analysis of amino acid changes in 72 samples showed 24 groups of samples with identical amino acid sequences of all six BKPyV proteins, namely three groups in subgroup IVc2, six in Ib1, ten in Ib2, one in Ia and four in subtype II. Identical amino acid sequences were found in more than two samples of subtype IVc2 and Ib1, and more than one such group of samples was present in subtype Ib2. For easier identification purposes, these groups of samples are further referred to as A (IVc2), B (Ib1) and C, D, E (Ib2) groups (Figure 3, Table 2).

**Figure 4:**
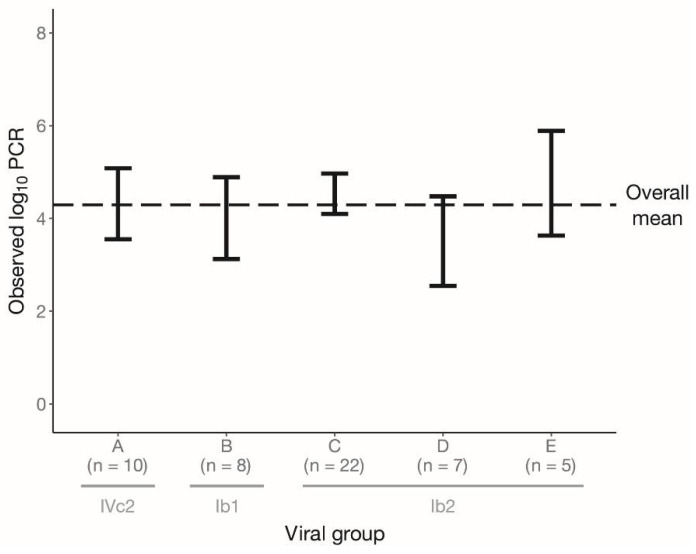
Distribution of the observed plasma BKPyV load values for A, B, C, D and E groups. The black error bars represent the mean ± (1.96× standard error) for each group. The black dashed line shows the mean of all plasma viral load observations belonging to these groups (*n* = 52). Viral load <2.7 log10 copies/mL was replaced by 1.35 log10 copies/mL.

We compared the mean of the plasma viral load of A (*n* = 10), B (*n* = 8), C (*n* = 22), D (*n* = 7) and E (*n* = 5) groups with the overall mean of the plasma viral load observed in samples of 52 patients but could not find a statistically significant difference for any of the groups (Figure 4; Table S3). The plasma viral load observed in samples of the remaining 20 patients can be found in Table A1.

### 3.4. BKPyV NCCR and Plasma Viral Load

In addition to the BKPyV coding region, we also analyzed the NCCR. The structure of the BKPyV NCCR was successfully determined in 147 samples: 45 plasma, 75 urine and 27 kidney biopsy samples (82 patients). The NCCR with an O-P-Q-R-S repeat block structure was detected in most of the samples (*n* = 136; 92.5%). The NCCR with a structure other than O-P-Q-R-S, or with incomplete blocks, was found in fewer plasma, urine and kidney biopsy samples (*n* = 11; Table 3). We also compared the BKPyV NCCR in a plasma sample with that in other samples from the same patients (*n* = 43) and found that the O-P-Q-R-S was the most common NCCR structure (*n* = 40; 93%). The low number of samples with rearranged NCCR did not allow us to further compare plasma viral load in patients to determine the possible association of high plasma viral load with the identified BKPyV NCCR patterns.

## 4. Discussion

In this retrospective study, we characterized the whole genome of BKPyV in early stage post-transplantation kidney biopsy, plasma and urine samples from a mono-center cohort of kidney recipients (University Hospitals Leuven, Leuven, Belgium). Our results revealed high intra-patient similarity of BKPyV genome sequences. This observation supports previous findings indicating that kidney allograft is the source of replicating BKPyV in kidney transplant recipients [11,35,36].

Our comparative analysis of the samples from the patients showed the predominance of subtype I, and more specifically subgroup Ib2, which accounted for 79.5% and 59%, respectively. The infection with subtypes IV (subgroup IVc2) and II was less common in our cohort and accounted for only 16.6% and 5.1%, respectively. These observations seem in line with previous studies where the Ib2 subgroup has been shown to be the most common BKPyV genetic variant in Europe [7].

Amino acid variations were observed in all six proteins of BKPyV. In addition, 13.6% of all amino acid positions in the Agno protein, 7.2% in VP1, 5.4%/6.5% in VP2/VP3, 3.2% in LTag, and 2.9% in sTag were found to be polymorphic. These results support previous findings indicating genetic variability in BKPyV detected by next-generation sequencing [37].

The comparative analysis of the entire BKPyV protein-coding regions obtained from the samples showed high intra-subgroup genetic diversity of the virus. We identified twenty-four viral groups with unique amino acid sequences in the five subgroups of BKPyV. Five of the twenty-four groups consisted of two and more samples: three groups within Ib2 and one in both Ib1 and IVc2. Although the analysis of plasma viral load did not reveal significant differences in the replication potential in any of the groups, our results nonetheless highlight the predominance of the aforementioned five BKPyV variants in patients. Worth mentioning is that the amino acid pattern in the BKPyV VP1 protein (at positions 340 and 362) has been described previously [5]. It has also been suggested that changes at these positions may influence the efficiency of the viral capsid assembly [5]. Further work is required to elucidate the exact role of the observed amino acid change patterns in the life cycle and pathogenesis of BKPyV.

We also checked the non-coding control region (NCCR) of BKPyV. Our analysis showed the prevalence of BKPyV with an archetypal structure (OPQRS) in plasma samples. This observation indicates that rearrangement of BKPyV NCCR is not necessary for the development of viremia. Due to the low number of BKPyV with rearranged NCCR in our patient samples, we were unable to investigate the association of the identified rearranged NCCR patterns and high plasma viral load in kidney transplant recipients at the early stages after kidney transplantation.

## 5. Conclusions

Our analysis of the entire BKPyV genome using next-generation sequencing revealed the presence of genetically distinct variants of BKPyV in kidney transplant recipients. The most common BKPyV genetic variant can be described as follows: BKPyV subgroup Ib2 with lysine at position 60 in the Agno protein, lysine and leucine at positions 340 and 362 in VP1, alanine at 341 in VP2 (222 in VP3) and the archetypal NCCR. The plasma viral load of none of the variants was significantly different from the overall mean observed at three months after kidney transplantation. Further validation in a larger cohort is warranted to better understand the link between the newly described BKPyV genetic variants and the development of viremia and PVAN in kidney transplant recipients.

## Figures and Tables

**Table 1:** BKPyV amino acid change distribution among samples collected at three months after kidney transplantation.

BKPyV Protein	Amino Acid Position	Amino Acid Change Per Position	Isolates, No. (%) (*n* = 127)	BKPyV VP1 Subtype/Subgroup	Group (Code)
Agno	14 15 29 43 56 57 58 59 60	VSLELPAVK	24 (18.9)	IVc2	1
		VSFELPAVK	21 (16.5)	Ib1	2
		VGFELPAVK	1 (0.8)	Ia	3
		LGFELPAVK	58 (45.7)	Ib2	4
		LGFEVxxxK	4 (3.1)	Ib2	5
		LGFKLPAVK	1 (0.8)	Ib2	6
		LGFELPAVR	13 (10.3)	Ib2	7
		IGFEVxxxK	5 (3.9)	II	8
sTag	28 36 78 97 143	PRSET	101 (79.5)	IVc2, Ib2, Ia	9
		PRSDT	20 (15.8)	Ib1	10
		SRSDT	1 (0.8)	Ib1	11
		PKNER	5 (3.9)	II	12
LTag	28 36 78 95 118 171 241 244 245 251 327 354 365 414 534 548 591 592 669 670 671 675	PRSRALDYIQTTDIPDAQRLDE	24 (18.9)	IVc2	13
		PRSSAQDHTQTTEIPDATRLDQ	4 (3.1)	Ib1	14
		PRSSAQDHTQTSEIPDATRSDQ	68 (53.5)	Ib2	15
		PRSSAQDHTQTTEIPDATRSDQ	15 (11.8)	Ib1	16
		PRSSAQDHTQTSEIPEATRSDQ	1 (0.8)	Ib2	17
		PRSSAEDHTQTSEIPDATRSDQ	1 (0.8)	Ib2	18
		PRSSAQDHTQTSEIPDATRSDE	4 (3.1)	Ib2	19
		PRSSAQDHTQTSEIPDAIRSDE	1 (0.8)	Ib2	20
		PRSSTQDHTQTSEIPDATRSDQ	1 (0.8)	Ib2	21
		SRSSAQDHTQTTEIPDATRSDQ	1 (0.8)	Ib1	22
		PRSSAQDHTQTTEIPDAARSDQ	1 (0.8)	Ib1	23
		PKNSAQDHIQITEVPDSKxxNQ	2 (1.6)	II	24
		PKNSAQDHIQITEVTDSKxxNQ	1 (0.8)	II	25
		PKNSAQDHIKITEVPDSKxxNQ	2 (1.6)	II	26
		PRSSAQEHTQITEIPDATRSDQ	1 (0.8)	Ia	27
VP1	42 60 61 62 66 69 71 73 74 75 77 82 83 117 138 139 145 175 178 210 225 284 316 340 353 362	VDNDYRTETADDRKENIEVIYPRQRV	20 (15.7)	IVc2	28
		VDNDYRTETAEDRKENIEVIYPRQRV	3 (2.4)	IVc2	29
		VDNDYKTETANDRKENIEVIYPRQRV	1 (0.8)	IVc2	30
		VDDNYKTENADDKKENVQIIYAKQRV	4 (3.1)	II	31
		VDDNYKTENADDKKQNVQIIYAKQRV	1 (0.8)	II	32
		LDENFKSENDSERQEHIEIIFARKKL	61 (48)	Ib2	33
		LDENFKSQNDSERQEHIEIIFARKKL	1 (0.8)	Ib2	34
		VDENFKSENDSERQEHIDIVFARRKL	19 (15)	Ib1	35
		VNENFKSENDSERQEHIDIVFARRKL	1 (0.8)	Ib1	36
		VDENFKSKNDSERQEHIDIVFARRKL	1 (0.8)	Ib1	37
		VDENFKSENDSERQEHIDVVFARRKL	1 (0.8)	Ia	38
		LDENFKSENDSDRQEHIEIIFARRKL	1 (0.8)	Ib2	39
		LDENFKSENDSERQEHIEIIFARRKV	13 (10.2)	Ib2	40
VP2 (VP3)	53 66 89 91 175(56) 209(90) 217(98) 221(102) 240(121) 242(123) 248(129) 256(137) 262(143) 263(144) 267(148) 268(149) 269(150) 318(199) 341(222)	SQTIAVDNQNRQMEKENQT	24 (18.9)	IVc2	41
		TQTIAIDNHNSEIEKESQT	3 (2.4)	II	42
		TQTIAMDNHNSEIEKESQT	1 (0.8)	II	43
		TQTIAVDNHNSEIEKESQT	1 (0.8)	II	44
		SQTIAIEDRHSEMDQQTKA	45 (35.3)	Ib2	45
		SQTIAIEDRHSEMDQQTKT	28 (22)	Ib2	46
		SETIAIEDRHSEMDQQTKT	3 (2.4)	Ib2	47
		SQTIAIEDRHSEMDQQTQT	19 (15)	Ib1	48
		SQTVAIEDRHSEMDQQTQT	2 (1.6)	Ib1	49
		SQSISIQDRHSEMDQQSQT	1 (0.8)	Ia	50

Amino acid numbering follows that of the Dunlop strain (V01108.1). VP2 protein contains four extra informative amino acid replacement sites in addition to those common to VP2 and VP3. A cross (x) indicates amino acid deletion.

**Table 2:** Amino acid sequences found in samples of BKPyV subgroups IVc2, Ib1 and Ib2.

BKPyV Subgroup	Group	Sample No.	Amino Acid Sequence (Code)				
			Agno	sTag	LTag	VP1	VP2/VP3
IVc2	A	10	VSL..…K (1)	…E. (9)	…R.L.YI..TD….Q.L.E (13)	V.NDYRT.TADD.K.N.EVIYP.QRV (28)	…..VDNQNRQ.EKENQT (41)
Ib1	B	8	VSF..…K (2)	…D. (10)	…S.Q.HT..TE….T.S.Q (16)	V.ENFKS.NDSE.Q.H.DIVFA.RKL (35)	…..IEDRHSE.DQQTQT (48)
Ib2	C	22	LGF..…**K** (4)	…E. (9)	…S.Q.HT..SE….T.S.Q (15)	L.ENFKS.NDSE.Q.H.EIIFA.**K**K**L** (33)	…..IEDRHSE.DQQTK**A** (45)
	D	7	LGF..…**K** (4)	…E. (9)	…S.Q.HT..SE….T.S.Q (15)	L.ENFKS.NDSE.Q.H.EIIFA.**R**K**V** (40)	…..IEDRHSE.DQQTK**T** (46)
	E	5	LGF..…**R** (7)	…E. (9)	…S.Q.HT..SE….T.S.Q (15)	L.ENFKS.NDSE.Q.H.EIIFA.**K**K**L** (33)	…..IEDRHSE.DQQTK**T** (46)

Amino acids that are identical among subgroups IVc2, Ib1 and Ib2 are represented by dots. Amino acids that distinguish the three groups in the Ib2 subgroup are shown in bold.

**Table 3:** Rearranged BKPyV NCCR in samples collected at three months after kidney transplantation.

NCCR Structure	Sample Type	Plasma Viral Load (log 10 copies/mL)	Access No.
OPQPQRS	kidney biopsy	N/A	OM938904
OPQPQRS	plasma	7.51	OM938905
OPPQRS	urine	N/A	OM938880
OPQRSRS	urine	N/A	OM938927
OPQQRS	urine	N/A	OM938944
OPPQRS	plasma	3.64	OM938858
OPPQRS	plasma	4.82	OM938920
OPPQRS	plasma	4.01	OM938941
OP(QR)S	kidney biopsy	N/A	OM938892
OP(Q)RS	urine	N/A	OM938835
OP(Q)RS	plasma	5.4	OM938834

Parentheses indicate a truncated NCCR block. N/A, not applicable.

## Data Availability

Please see Section 2.

## Supplementary Materials

The following supporting information can be downloaded at https://www.mdpi.com/article/10.3390/v14071533/s1: Figure S1: Schematic illustration of a BKPyV genome with six regions encoding the Agno, VP1, VP2, VP3, LTag and sTag proteins and the location of the two set of primers that were used to generate two amplicons (amplicon 1 and 2); Table S1: Primer sequences; Table S2: Estimation of difference between the average viral load in each subtype/subgroup and the overall mean; Table S3: Estimation of difference between the average viral load in A, B, C, D and E groups and the overall mean.

## Appendix A

**Table A1:** BKPyV amino acid diversity and plasma viral load detected in samples from 72 patients three months after transplantation.

BKPyV Subtype/Subgroup	Group	Sample, No. (*n* = 72)	Plasma Viral Load (log10 copies/mL)	Amino Acid Sequence (Code)				
				Agno	sTag	LTag	VP1	VP2/VP3
IVc2 (*n* = 12)	A	10	4.31 *	VSLELPAVK (1)	PRSET (9)	PRSRALDYIQTTDIPDAQRLDE (13)	VDNDY**R**TETA**D**DRKENIEVIYPRQRV (28)	SQTIAVDNQNRQMEKENQT (41)
	N/A	1	5.42	……... (1)	….. (9)	…………………. (13)	…..R….E…………... (29)	………………. (41)
	N/A	1	5.76	……... (1)	….. (9)	…………………. (13)	…..K….N…………... (30)	………………. (41)
Ib1 (*n* = 13)	B	8	4 *	VSFELPAVK (2)	**P**RSDT (10)	**P**RSSAQDHTQTTEIPDA**T**R**S**DQ (16)	V**D**ENFKS**E**NDSERQEHIDIVFARRKL (35)	SQT**I**AIEDRHSEMDQQTQT (48)
	N/A	1	3.16	……... (2)	….. (10)	……………..A…. (23)	…………………….. (35)	………………. (48)
	N/A	1	3.79	……... (2)	….. (10)	……………….L.. (14)	…………………….. (35)	………………. (48)
	N/A	1	3.1	……... (2)	….. (10)	……………….L.. (14)	…………………….. (35)	...V…………... (49)
	N/A	1	4.97	……... (2)	….. (10)	…………………. (16)	…….K……………... (37)	………………. (48)
	N/A	1	3.47	……... (2)	S…. (11)	S………………... (22)	.N…………………... (36)	………………. (48)
Ib2 (*n* = 42)	C	22	4.53 *	LGF**ELPAVK** (4)	PRSET (9)	PRSS**AQ**DHTQTSEIP**D**A**T**RSD**Q** (15)	LDENFKS**E**NDS**E**RQEHIEIIFAR**K**K**L** (33)	S**Q**TIAIEDRHSEMDQQTK**A** (45)
	D	7	3.51 *	LGF**ELPAVK** (4)	PRSET (9)	PRSS**AQ**DHTQTSEIP**D**A**T**RSD**Q** (15)	LDENFKS**E**NDS**E**RQEHIEIIFAR**R**K**V** (40)	S**Q**TIAIEDRHSEMDQQTK**T** (46)
	E	5	4.76 *	LGF**ELPAVR** (7)	PRSET (9)	PRSS**AQ**DHTQTSEIP**D**A**T**RSD**Q** (15)	LDENFKS**E**NDS**E**RQEHIEIIFAR**K**K**L** (33)	S**Q**TIAIEDRHSEMDQQTK**T** (46)
	N/A	2	4.64 *	...EVxxxK (5)	….. (9)	………………...E (19)	…………………….. (33)	………………. (46)
	N/A	1	2.85	……... (4)	….. (9)	…………...E…... (17)	…………………….. (33)	………………. (45)
	N/A	1	<2.7	……... (4)	….. (9)	……………..I...E (20)	…………………….. (33)	………………. (45)
	N/A	1	<2.7	……... (4)	….. (9)	………………….(15)	………..D………..R.L (39)	………………. (46)
	N/A	1	4.12	...KLPAVK (6)	….. (9)	…..E……………. (18)	…….Q…………...K.L (34)	………………. (45)
	N/A	1	5.7	……... (7)	….. (9)	………………….(15)	…………………….. (33)	.E…………….T (47)
	N/A	1	3.64	……... (4)	….. (9)	….T…………….. (21)	…………………….. (33)	………………. (45)
Ia (*n* = 1)	N/A	1	2.78	VGFELPAVK (3)	PRSET (9)	PRSSAQEHTQITEIPDATRSDQ (27)	VDENFKSENDSERQEHIDVVFARRKL (38)	SQSISIQDRHSEMDQQSQT (50)
II (*n* = 4)	N/A	1	5.06	IGFEVxxxK (8)	PKNER (12)	PKNSAQDHI**Q**ITEV**P**DSKxxNQ (24)	VDDNYKTENADDKK**E**NVQIIYAKQRV (31)	TQTIA**I**DNHNSEIEKESQT (42)
	N/A	1	3.89	IGFEVxxxK (8)	PKNER (12)	PKNSAQDHI**Q**ITEV**P**DSKxxNQ (24)	VDDNYKTENADDKK**E**NVQIIYAKQRV (31)	TQTIA**M**DNHNSEIEKESQT (43)
	N/A	1	4.63	IGFEVxxxK (8)	PKNER (12)	PKNSAQDHI**K**ITEV**P**DSKxxNQ (26)	VDDNYKTENADDKK**E**NVQIIYAKQRV (31)	TQTIA**I**DNHNSEIEKESQT (42)
	N/A	1	<2.7	IGFEVxxxK (8)	PKNER (12)	PKNSAQDHI**Q**ITEV**T**DSKxxNQ (25)	VDDNYKTENADDKK**Q**NVQIIYAKQRV (32)	TQTIA**V**DNHNSEIEKESQT (44)

* An average plasma viral load observed in ≥2 samples (plasma viral load of <2.7 log10 copies/mL were replaced by 1.35 log10 copies/mL). Identical amino acids among samples of subgroup IVc2, Ib1 and Ib2 are represented by dots. Amino acids that distinguish samples of the same subtype/subgroup are shown in bold. A cross (x) indicates amino acid deletion.
