# Supplementary material for: Polyomavirus BK Genome Comparison Shows High Genetic Diversity in Kidney Transplant Recipients Three Months after Transplantation
Source: Viruses. 2022 Jul 14;14(7):1533. doi: 10.3390/v14071533 (PMC9318200; doi:10.3390/v14071533)
Supplement: Supplementary file 1 [file viruses-14-01533-s001.zip › Figure S1.pdf]

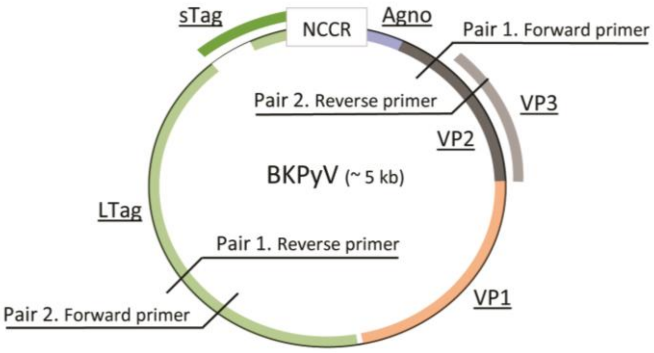

Figure S1. Schematic illustration of a BKPyV genome with six regions en-coding the Agno, VP1, VP2, VP3, LTag and sTag proteins and the location of the two set of primers that were used to generate two amplicons (amplicon 1 and 2).
