# Supplementary material for: Polyomavirus BK Genome Comparison Shows High Genetic Diversity in Kidney Transplant Recipients Three Months after Transplantation
Source: Viruses. 2022 Jul 14;14(7):1533. doi: 10.3390/v14071533 (PMC9318200; doi:10.3390/v14071533)
Supplement: Supplementary file 1 [file viruses-14-01533-s001.zip › Table S1.pdf]

Table S1. Primer sequences

Target	Primer		Sequence (5' - 3')
BKPyV genome	Pair 1	Forward	CTATTGCTGGGTTTGCTGCT
		Reverse	AGAACCCAAATATTTCCACCAGG
	Pair 2	Forward	CAGTGCTTGATCCATGTCCAG
		Reverse	GGCATTTACAATTGTCCAGGTAG
JCPyV genome	Pair 1	Forward	GTAGCTGGGTTTGCTGCATTGG
		Reverse	ACAGGTGTTTCCACCTGGAATT
	Pair 2	Forward	CCATGTCCAGAGTCTTCTGCT
		Reverse	TGGTGAATTAACTATTGCCCAAG
BKPyV NCCR		Forward	GGCAAAGAGGAAAATCAGCACA
		Reverse	CAGCACCCATGAACCTGGAA

BKPyV, BK polyomavirus. JCPyV, JC polyomavirus. NCCR, non-coding control region.
