# Supplementary material for: Polyomavirus BK Genome Comparison Shows High Genetic Diversity in Kidney Transplant Recipients Three Months after Transplantation
Source: Viruses. 2022 Jul 14;14(7):1533. doi: 10.3390/v14071533 (PMC9318200; doi:10.3390/v14071533)
Supplement: Supplementary file 1 [file viruses-14-01533-s001.zip › Table S2.pdf]

Table S2. Estimation of difference between the average viral load in each subtype/subgroup and the overall mean

Variable	Estimate	Standard error	P Value
Intercept	4.04	0.17	< 0.001
Ia	-1.26	1.43	0.38
Ib1	-0.37	0.34	0.28
Ib2	0.09	0.14	0.52
II	-0.17	0.72	0.81
IVc2	0.26	0.37	0.49

The intercept equals the mean of all observed viral loads across the patients with different subtypes/subgroups.
