# Supplementary material for: Polyomavirus BK Genome Comparison Shows High Genetic Diversity in Kidney Transplant Recipients Three Months after Transplantation
Source: Viruses. 2022 Jul 14;14(7):1533. doi: 10.3390/v14071533 (PMC9318200; doi:10.3390/v14071533)
Supplement: Supplementary file 1 [file viruses-14-01533-s001.zip › Table S3.pdf]

Table S3. Estimation of difference between the average viral load in A, B, C, D and E groups and the overall mean

Variable	Estimate	Standard error	P Value
Intercept	4.29	0.19	< 0.001
A	0.02	0.39	0.95
B	-0.29	0.45	0.52
C	0.24	0.22	0.28
D	-0.78	0.49	0.11
E	0.47	0.58	0.42

The intercept equals the mean of all observed viral loads across the patients with different viral groups.
